# Using Social Media and Web-Based Networking in Collaborative Research: Protocol for the Geriatric Medicine Research Collaborative

**DOI:** 10.2196/resprot.9304

**Published:** 2018-10-09

**Authors:** Geriatric Medicine Research Collaborative

**Affiliations:** 1 Institute of Inflammation and Ageing College of Medical and Dental Sciences University of Birmingham Birmingham United Kingdom

**Keywords:** collaborative, geriatrics, social media, virtual communication, trainee-led

## Abstract

**Background:**

Traditional pathways to promote research collaboration typically take years to expand beyond individual institutions. Social media and online networking provide an innovative approach to promote research collaboration.

**Objective:**

The objective of this paper is to present the formation of the Geriatric Medicine Research Collaborative, United Kingdom — a national trainee-led research collaborative. This collaborative aims to facilitate research projects that will directly benefit older patients, improve research skills of geriatric medicine trainees, and facilitate recommendations for health care policy for older adults.

**Methods:**

Our methods of collaboration comprised trainee-led meetings regionally and at national conferences, email communication, direct uploading of project material to our website, social media, and virtual meetings. Structured use of local, regional, and network leads has facilitated this collaboration. Having a clear virtual presence has been the key to the rapid development of the network.

**Results:**

The use of social media and online networking encouraged the involvement of multiple regions early in the development of the collaborative and allowed rapid dissemination of project ideas. This facilitated the collection of large datasets and enhanced scientific validity of project outcomes. Furthermore, this has the potential to transform geriatric medicine research, as older patients have been historically excluded from large commercial trials due to multimorbidity, frailty, and cognitive impairment.

**Conclusions:**

Perceived limitations to predominantly online or virtual collaboratives, including reduced accountability, and loss of interpersonal relationships are balanced by increased trainee engagement, high frequency of communication, and rapid access to a breadth of expertise. Utilization of virtual communication has the potential to lead to future interspecialty, interprofessional, and international collaboration, and to accelerate research that improves outcomes for older adults.

## Introduction

### Background

Over the last 10 years, trainee-led research collaboratives have been established within the United Kingdom and internationally [1,2]. An example includes the general surgical research collaborative, which began regionally as the West Midlands Research Collaborative and has since led to the development of national and international surgical collaborations [3,4]. Traditional pathways to promote collaboration between researchers can take years to expand beyond individual institutions and regions. The traditional collaboration relied on networking events and meetings, which were limited to geographically accessible areas, chance meetings, or expensive trips. Social media and online networking provide an innovative approach to the development and promotion of a research collaborative, with the potential to expand instantly and link otherwise unconnected individuals. We have utilized this approach in the formation of the “Geriatric Medicine Research Collaborative (GeMRC)” in the United Kingdom.

Collaboration has long been emphasized as an important aspect of academic development [5]. Traditional pathways of academic medicine have focused on the development of independent researchers [6,7]. While this may result in high-caliber researchers on an individual level, this approach may be detrimental to the academic progress overall; in grant development stages, independent researchers may be reluctant to share their niche of expertise with those outside of their organization for fear of affecting their own career progression [8]. In addition, due to the perceived skills and infrastructure required to undertake research with high patient benefit, such as a randomized controlled trial (RCT), individuals may be reluctant to undertake this early in their career. Furthermore, having a collaborative network ameliorates this trepidation.

Considering the aging population and disparate health and social care needs of older adults [9], the National Institute for Health Research (NIHR) recently released a themed call for research involving “Older adults with complex needs” [10]. Although geriatric medicine is a popular medical specialty, engagement with academic geriatric medicine has previously been less popular than other academic pathways [11]. There are a number of reasons for this finding. First, clinicians who typically choose a career in geriatric medicine tend to be more interested in direct patient care [12]. Second, there is a limited drive within the trainee curriculum to achieve research competence [13]. The majority of trainee time is spent in clinical areas, and, often, there are service requirements related to high rates of complex inpatients. In addition, the problem tends to cycle as trainers have limited experience of research to facilitate training of the next generation. Unfortunately, older adults have historically been excluded from research trials due to frailty, multimorbidity, and cognitive impairment [14], and results of trials undertaken in younger adults may not be generalizable [15,16]. Furthermore, engagement of geriatric medicine trainees in research may help increase translational research and, thus, lead to improvements in the care of this vulnerable population.

### Aims and Objectives of Geriatric Medicine Research Collaborative

The following are the aims and objectives of GeMRC:

1. To enable the prompt conduct of research projects that are likely to have a direct impact on patient care upon completion.
2. To enable trainees in geriatric medicine to develop valuable research skills while undertaking their clinical training.
3. To obtain a clearer understanding of health care provision by conducting multisite audit and quality improvement projects to enable clearer recommendations for health care policy.

## Methods

### Development and Organization

GeMRC has been completely trainee-led from creation, through to project idea generation, and conduct of audit and research projects. Our initial dissemination involved creation of national [17] and regional websites [18], emphasizing the success of other research collaboratives. This approach encouraged the engagement of trainees, including those with minimal research experience. Our first meeting led to the generation of original ideas, which were subsequently presented to and discussed with other research collaboratives and the NIHR Clinical Research Network (CRN).

GeMRC rapidly expanded from a single region to a national collaborative involving 14 out of a potential 15 regions within the United Kingdom within 3 months of creation; the use of online dissemination of our collaborative was greatly beneficial toward this. GeMRC was advertised through trainee bulletins, discussion on general geriatric medicine websites [19], Twitter [20], and through contacting trainees directly via email to invite participation. This trainee-led grassroots approach encouraged early participation. Social media continues to be pivotal in encouraging engagement of trainees, other specialties, and nonmedical individuals. Additional social media networks including LinkedIn [21], Facebook [22], YouTube [23], and Periscope [24] have since been utilized. The use of general hashtags such as “geriatrics” and specific project-related hashtags such as “delirium” enables rapid dissemination to stakeholders.

Although Twitter has proven to be an efficient tool to engage with the academic community, additional social media networks have provided added benefits. The promotion of our collaborative and research projects using Facebook has encouraged engagement with trainees who have been previously less involved with academia; although Twitter has been commonly used within the academic community, usage beyond this has been limited by many trainees. Of note, Facebook has broadened our audience. LinkedIn has been beneficial in engaging with nonmedical individuals. We utilized YouTube to publish a PowerPoint presentation in relation to our most recent project, “Delirium Day Audit,” discussed below. Similarly, Periscope is a social media outlet that offers live streaming; this was used to offer a live question and answer session prior to the audit.

One of the pivotal concepts of our research collaborative is that all members and researchers are considered equal. All publications will be published under the name of the GeMRC. All data collectors will be formally acknowledged as collaborators for authorship purposes for all of our studies. Specific trial and study steering groups will be listed separately to other collaborators as appropriate. Our approach to contribution is flexible, allowing trainees to be involved in the process of study design, obtaining ethical approval, public involvement, participant recruitment, analysis of results, and dissemination. Trainees may choose to be involved in all or part of these processes. However, in order to formalize communication, we have developed a formal structure, described below.

### Network Leads

The responsibility of the network leads is to oversee the GeMRC overall and to ensure regional communication and training of regional leads. This is currently the responsibility of the founding members. It is envisaged that over time, this role will rotate through trainees nationally. Network leads communicate with regional leads through a combination of email, WhatsApp, social media, and virtual meetings. WhatsApp has been especially useful in providing instant communication responses. Small virtual meetings were initially conducted with appear.in (free plan) [25]. The videoconferencing platform Zoom is now being used to enable larger meetings with all regional representatives [26]. These are conducted on weekday evenings on prearranged dates, at least four times each year; more frequent meetings may be organized in addition to these. The NIHR CRN Ageing group has recently developed two trainee representative roles. These trainees have formal involvement in GeMRC, along with the British Geriatrics Society (BGS) Research and Academic Development trainee representative; this has been invaluable in achieving support from the NIHR CRN Ageing group and the BGS, and senior academic geriatricians nationally.

### Regional Leads

Regions within the United Kingdom have been divided according to the boundaries of regional training programs; this includes collaboration with the Welsh Geriatrician’s Network [27]. This ensures ease of communication with trainees through regional trainee representatives for geriatric medicine training. The regional lead for GeMRC does not need to be the regional trainee representative, but he or she should maintain regular communication with the trainees to ensure that all trainees within the region are kept up to date about projects and GeMRC progress. In addition, the regional lead communicates with local leads about individual projects and arranges regional meetings as appropriate; this occurs through a combination of emails, website updates, and WhatsApp. Furthermore, regional leads are responsible for training local leads. Notably, the regional lead communicates with other regions as necessary through the network leads.

### Local Leads

While we accept that not all trainees will wish to participate in all projects, we aim to have one local representative for GeMRC in each hospital trust. Local leads are responsible for the site conduct of all projects and local data collection. In addition, local leads are responsible for engaging consultants in our projects; working with local key stakeholders such as head of departments, specialist nurses, and allied health care professionals. Local leads also provide training to other trainees working locally. The local lead is required to communicate regularly with his or her regional lead through emails, website updates, WhatsApp, and virtual and in-person meetings.

### Patient and Public Involvement

Individuals aged above 70 years are the fastest growing users of social media [28]. We intend to harness this by communicating our research on Twitter, LinkedIn, YouTube, and Periscope in terms understandable to nonmedical professionals. We have created a website specifically for the purpose of relaying the rationale, design, and findings of our research to nonmedically trained individuals [29]. Concurrently, we will use our national website and social media to facilitate the organization of regional discussion group meetings involving older adults and their carers.

### Journal Club

Virtual journal clubs have recently grown in popularity. In contrast to traditional in-person journal clubs, virtual discussions enable those involved to read and critique papers in their own time and comment remotely. The most common method for conducting virtual journal clubs is to utilize social media. While this has the benefit of enabling a broad audience, comments and messages may be limited and the involvement may be time-dependent to prevent interspersion with comments pertaining to other topics. We have incorporated a membership function into our national website to enable the organization of a national journal club through a forum [30]. Files are uploaded directly to the forum. Members can review all files and comments in their own time and provide their own critique of journal papers. Although a national rota is created to participate in this, we have a flexible approach to involvement.

## Results

### Current Projects

The projects detailed below are all currently underway or in development. We plan to publish the protocol for our research projects in peer-reviewed journals so that these are widely available. The results of all of our projects will be presented at national conferences and published in peer-reviewed journals under the name of GeMRC.

#### Collaborative Research Projects

##### Ferric Carboxymaltose to Prevent Blood Product Use Following Operative Management of Neck of Femur Fractures

This RCT will assess the effect of ferric carboxymaltose on the postoperative prescription of packed red cells compared with standard care. In addition, secondary outcomes including the length of stay, mortality, and delirium incidence will be recorded. This trial will be supported by the Birmingham Clinical Trials Unit. We are currently in the process of applying for funding, initially through the NIHR Research for Patient Benefit funding program. The protocol for this trial will be published and widely available in the future.

##### Chlorhexidine Mouthwash to Prevent Hospital-Acquired Pneumonia in Older Hospital Inpatients

This RCT will assess the impact of chlorhexidine mouthwash on the incidence of hospital-acquired pneumonia. The protocol for this study is currently under development and will be published when finalized by our steering group. To ensure that this is a cost-effective study that can be conducted at scale, we will use a before- during- and after- intervention analysis.

#### Collaborative Audit and Service Evaluation Projects

##### Perioperative Management of Anaemia in Patients With Fractured Necks of Femurs

This project aimed to assess the current practice of perioperative management of anemia in patients undergoing operative management of the fractured necks of femurs across multiple hospital sites using retrospective electronic and paper notes assessment. Current management was assessed against agreed standards adapted from British Orthopaedic Society guidelines [31] and the National Institute for Health and Clinical Excellence blood transfusion guidelines [32]. This project was publicized through our website, emails, and Twitter. In addition, related documents were uploaded to our website for direct downloads. Seven sites participated in this initial study. Notably, results have been presented at the national BGS Spring Meeting 2018 and have assisted in providing preliminary data toward our proposal for the FErric carboxymaltose to preveNt blood proDuct use following Operative management of neck of Femur Fractures (FEND-OFF) study.

##### Evaluation of Current Practice of Mouth Care Amongst Older Medical Inpatients

This service evaluation aimed to assess the current standards of mouth care that older adults receive when admitted to hospital. Our methodology incorporated a 1-day flash evaluation of mouth care in hospitalized patients and a survey of relevant knowledge among UK doctors. We uploaded study-related documents to the website and disseminated the information via email. Our survey was hosted on Google Forms [33], and the link was disseminated via emails and Twitter. In the flash audit part of this study, 15 sites participated. We obtained 136 responses to our survey. Results have been presented at the National Spring BGS Meeting 2018 and will guide further multisite quality improvement projects and our proposal for the Chlorhexidine mOUthwash to preveNt hospiTal-acquired pnEumonia in oldeR hospital inpatients (COUNTER) study.

##### “Delirium Day Audit”: Evaluation of Delirium Assessment and Recognition in Acutely Hospitalized Older Adults

This national audit was conducted on the World Delirium Day on Wednesday, March 14, 2018. Overall, 67 sites registered to participate in this audit, and results are currently being analyzed. In the United Kingdom, the National Institute for Health and Clinical Excellence guidelines recommend that all adults aged ≥65 years newly admitted to a hospital should be screened for delirium [34]. However, this is not always performed in practice; delirium remains underrecognized in many cases [35,36]. This study evaluated whether older patients had been assessed for delirium and whether delirium had been recognized. In addition, secondary data analysis was performed on the anonymized database to determine the point prevalence of delirium. We publicized our study through our website, WhatsApp, emails, Twitter, Facebook, and LinkedIn. Study-related documents were uploaded to our website, and a Google Docs spreadsheet was used to record participation at each site. Furthermore, a PowerPoint presentation to clarify the audit process was uploaded to YouTube, a live Web stream question and answer session was hosted on Periscope, and videos demonstrating delirium assessment using real patients were uploaded to a password-protected part of our website [37]. Local quality improvement projects are currently underway, and we will conduct a national reaudit later this year.

### Funding

We have described the above processes for applying for research grants for specific projects. In the same way that all collaborators who contribute toward projects are acknowledged in authorship, we have agreed on a policy that all collaborators who contribute toward project development should be listed on grant applications. The initial set-up of GeMRC was free. We utilized our own skills in website development using the free Wix server and created our own logo using Paint 3D (Microsoft Corporation, United Kingdom), which has now become highly recognizable. Our logo has been incorporated into our Google, YouTube, Twitter, Facebook, LinkedIn, and Periscope accounts and also added to our email signature and WhatsApp group. In addition, we used free teleconference software as described. However, we anticipate that there will be ongoing costs related to the management of GeMRC. We have successfully obtained funding from a West Midlands BGS grant to cover regional and national networking costs. All West Midlands members were listed on our initial regional grant. This has been used initially to purchase the domain name for our website and remove Wix adverts. This has improved the credibility of our collaborative and improved the ease to locate it online. Further funding will be used for patient and public involvement activities.

## Discussion

Trainee-led research collaboratives, driven by online networking and social media, are an innovative approach to conducting national audit and research projects. The use of social media and online networking allows rapid dissemination of project ideas and involvement of multiple regions early in the development of a collaborative. This facilitates the collection of much larger datasets and enhances the scientific validity of project outcomes. A particularly innovative approach has been to create a separate website specifically for the purpose of communicating our research ideas, project design, and results to nonmedically trained individuals.

Many older adults have complex needs. Conventional research may be less applicable to this group of patients; they are often underrecruited in studies or excluded because of their comorbidities [14]. Historically, there has been minimal emphasis on research within the geriatric medicine curriculum [13]. This can be considered both a positive and negative aspect. While the removal of coerce improves morale and enthusiasm of those undertaking research, the lack of organized structure and opportunities may reduce involvement in research. A lack of research infrastructure in geriatric medicine may have reduced the opportunity for involvement of trainees in research and subsequent retention in academic careers in geriatric medicine. GeMRC offers an opportunity to ensure rapid conduct of research projects from the generation of ideas to completion, with subsequent early implementation of changes into clinical practice to improve patient care.

There are limitations to a purely online research collaborative. Purely written electronic communication can lead to misinterpretation of concepts, less-developed interpersonal relationships, and reduced accountability. However, this is countered by the ease of communication and the ability to arrange virtual meetings at short notice, without expensive and time-consuming travel. In addition, email correspondence provides a clear written record of exactly when project timelines are planned and who is responsible for each stage. We believe online networking has the potential to change the way clinical geriatric research is conducted; this will benefit the trainees involved, improve patient outcomes, and shape the academic medicine of the future.

## Abbreviations

BGS: British Geriatrics Society
COUNTER: chlorhexidine mouthwash to prevent hospital-acquired pneumonia in older hospital inpatients
CRN: Clinical Research Network
FEND-OFF: ferric carboxymaltose to prevent blood product use following operative management of neck of femur fractures
GeMRC: Geriatric Medicine Research Collaborative
NIHR: National Institute for Health Research
RCT: randomized controlled trial
